# A clinical trial termination prediction model based on denoising autoencoder and deep survival regression

**DOI:** 10.1002/qub2.43

**Published:** 2024-04-12

**Authors:** Huamei Qi, Wenhui Yang, Wenqin Zou, Yuxuan Hu

**Affiliations:** ^1^ School of Electronic Information Central South University Changsha Hunan China; ^2^ School of Computer Science and Engineering Central South University Changsha Hunan China

**Keywords:** clinical trials, denoising autoencoder, DeepSurv, experimental termination, survival analysis

## Abstract

Effective clinical trials are necessary for understanding medical advances but early termination of trials can result in unnecessary waste of resources. Survival models can be used to predict survival probabilities in such trials. However, survival data from clinical trials are sparse, and DeepSurv cannot accurately capture their effective features, making the models weak in generalization and decreasing their prediction accuracy. In this paper, we propose a survival prediction model for clinical trial completion based on the combination of denoising autoencoder (DAE) and DeepSurv models. The DAE is used to obtain a robust representation of features by breaking the loop of raw features after autoencoder training, and then the robust features are provided to DeepSurv as input for training. The clinical trial dataset for training the model was obtained from the ClinicalTrials.gov dataset. A study of clinical trial completion in pregnant women was conducted in response to the fact that many current clinical trials exclude pregnant women. The experimental results showed that the denoising autoencoder and deep survival regression (DAE‐DSR) model was able to extract meaningful and robust features for survival analysis; the C‐index of the training and test datasets were 0.74 and 0.75 respectively. Compared with the Cox proportional hazards model and DeepSurv model, the survival analysis curves obtained by using DAE‐DSR model had more prominent features, and the model was more robust and performed better in actual prediction.

## 1 INTRODUCTION

Effective clinical trials are necessary for the treatment, diagnosis and understanding of medical advances [1]. Due to the life‐threatening nature of certain diseases (e.g., chronic or acute) and unmet medical needs, and due to surprises (e.g., poor treatment outcomes or excessive drug toxicity), trials may be terminated early [2]. Despite the full financial and institutional support of national agencies such as the National Science Foundation (NSF) and the National Institute of Health (NIH), termination of studies is still very common. It has been reported that approximately 19% of all studies registered on the clinicaltrials.gov repository were terminated before they produced results [3]. Early termination without reaching a conclusion can have significant financial, ethical and scientific implications [4]. To reduce the loss of human, material and financial resources, the survival analysis model is applied to predict the completion of clinical trials in advance. If the probability of early termination is high, the trial protocol can be adjusted in advance, according to the experimental parameters that affect the probability of termination, in order to effectively avoid some termination situations.

For ethical reasons and because of the specific nature of the pregnant patient population, the majority of premarketing drug clinical trials cannot be conducted on pregnant women [5], so that many clinical trials exclude trials on pregnant women. A study by Shields et al. indicated that of 367 phase IV trials with validated inclusion and exclusion criteria, 348 (95%) excluded pregnant women [6]. However, for most drugs, the risk of minor teratogenicity or more subtle effects on fetal development remains to be determined [5]. It is therefore crucial to anticipate the feasibility of clinical trial protocols in the pregnant population well in advance of the study planning phase.

To predict the completion of clinical trial protocols in the pregnant population, some researchers have used the Cox proportional hazards model (CPH) [7, 8], a regression model commonly used in survival analysis. Kim et al. used CPH and DeepSurv to predict the completion of clinical trials for pregnant women and compared the predictive performance of the two methods, both achieving a C‐index of 0.72 [9]. However, in clinical trial studies, One‐Hot Encoding (binary pattern) is used to represent each feature in order to easily capture information specific to an individual experiment [10]. As the number of features to be represented in a clinical trial dataset increases, the feature space becomes sparse (with many zeros) [11]. When the model is trained, the features are too fine‐grained, which can lead to overfitting of the trained model, making it less accurate and less robust. In the training of the DeepSurv model by Kim et al. they did not consider the sparsity of the features in the clinical trial dataset and trained for all features in the dataset, which may lead to higher training accuracy than testing accuracy [9].

To overcome the problem of model overfitting due to sparse features in clinical trial datasets, Wang et al., used the Denoising Autoencoder (DAE) to extract meaningful and more robust features related to survival prediction from lung adenocarcinoma (ADC) data [12]. The DAE model is a stochastic version of the autoencoder technique [12]. The DAE trains the autoencoder to reconstruct clean (repaired) inputs from partially corrupted (damaged) inputs to force the hidden layer of the autoencoder to capture more robust features [13].

To address the problems of clinical trial completion prediction and sparse features in clinical trial datasets, we combined the DAE model with the DeepSurv model for clinical trial completion prediction, considering that the DeepSurv model of Kim et al. [9] is less effective than the DAE model of Wang et al. [12] for datasets with sparse features.

The aim of the study is to propose a model based on denoising autoencoder and deep survival regression (DAE‐DSR) for predicting clinical trial completion in pregnant women. A dataset of clinical trials of pregnant women with a large number of features and a sparse feature space was collected from ClinicalTrials.gov. The DAE model was applied to analyze this large dataset by inputting sparse or corrupted features to reconstruct the features of the original data in order to improve the accuracy and robustness of the model.

The main contributions of this paper are: (1) consideration of the problem of model overfitting due to sparse clinical datasets, by introducing DAE in order to extract more meaningful and robust features related to survival prediction, (2) provision of important experimental parameters that affect the probability of clinical trial termination to facilitate advanced adjustment of clinical trial protocols by subsequent researchers. The rest of the paper is organized as follows: Section 2 presents the results of experiment, Section 3 reviews previous research on CPH and autoencoders, Section 4 is the conclusion, and finally Section 5 describes the methodology of the paper.

## 2 RESULTS

### 2.1 Model performance comparison

Table 1 shows the experimental results from our proposed model and compares the performance with the Cox proportional risk model and the DeepSurv model. DeepSurv is closely related to our approach, which uses a double hidden layer multilayer perceptron (MLP) for the survival analysis network.

**TABLE 1:** Comparison of model performance (C‐index) between different survival analysis methods in the training set and test set.

Approach	Training dataset	Test dataset
CPH	0.72	0.71
DeepSurv (CoxPH)	0.73	0.70
DAE‐DSR	0.74	0.75

Abbreviations: CPH, Cox proportional hazard model; C‐index, concordance index; DAE‐DSR, denoising autoencoder and deep survival regression.

**FIGURE 1:**
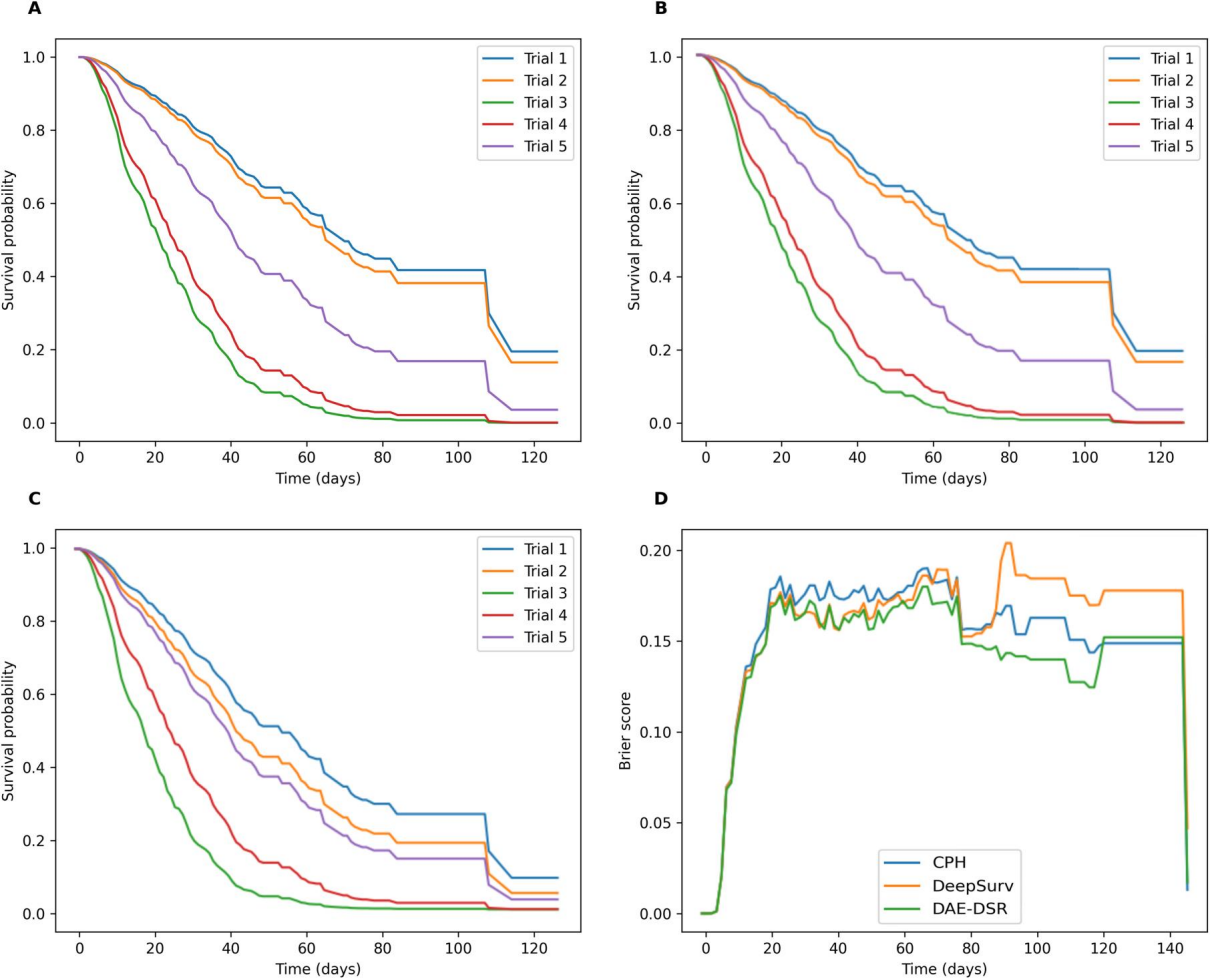
Survival probability prediction and Brier score in the test set. (A–C) are survival curves for five clinical trial completions with trials 1, 2, 3, 4, and 5 for the Cox proportional hazards model, DeepSurv, and denoising autoencoder and deep survival regression models, respectively; and (D) shows the BS over time of the three models. BS, Brier score.

Figure 1A–C show the survival curves for each of the first five clinical trials in the test set for each of the three models, CPH, DeepSurv, and DAE‐DSR models, respectively. Compared to the first two models, the survival analysis curves under the DAE‐DSR model have prominent features and greater model robustness. The proposed model offers better estimates of the probability distributions, and in most cases, the predicted probabilities provided by the CPH model and DeepSurv are much worse than those from the DAE‐DSR calibration; Figure 1D shows the Brier score (BS) over time for the three models.

The comparison shows that the non‐linear model using neural networks(DeepSurv, DAE‐DSR) has better performance than the original linear model(CPH). From Table 1, the DAE‐DSR model is better at solving the overfitting problem, while providing prediction accuracy. This is because, before training, we added noise to the dataset to simulate the sparse effects of real data, allowing the encoder to obtain more robust feature representation.

### 2.2 Feature impact

**FIGURE 2:**
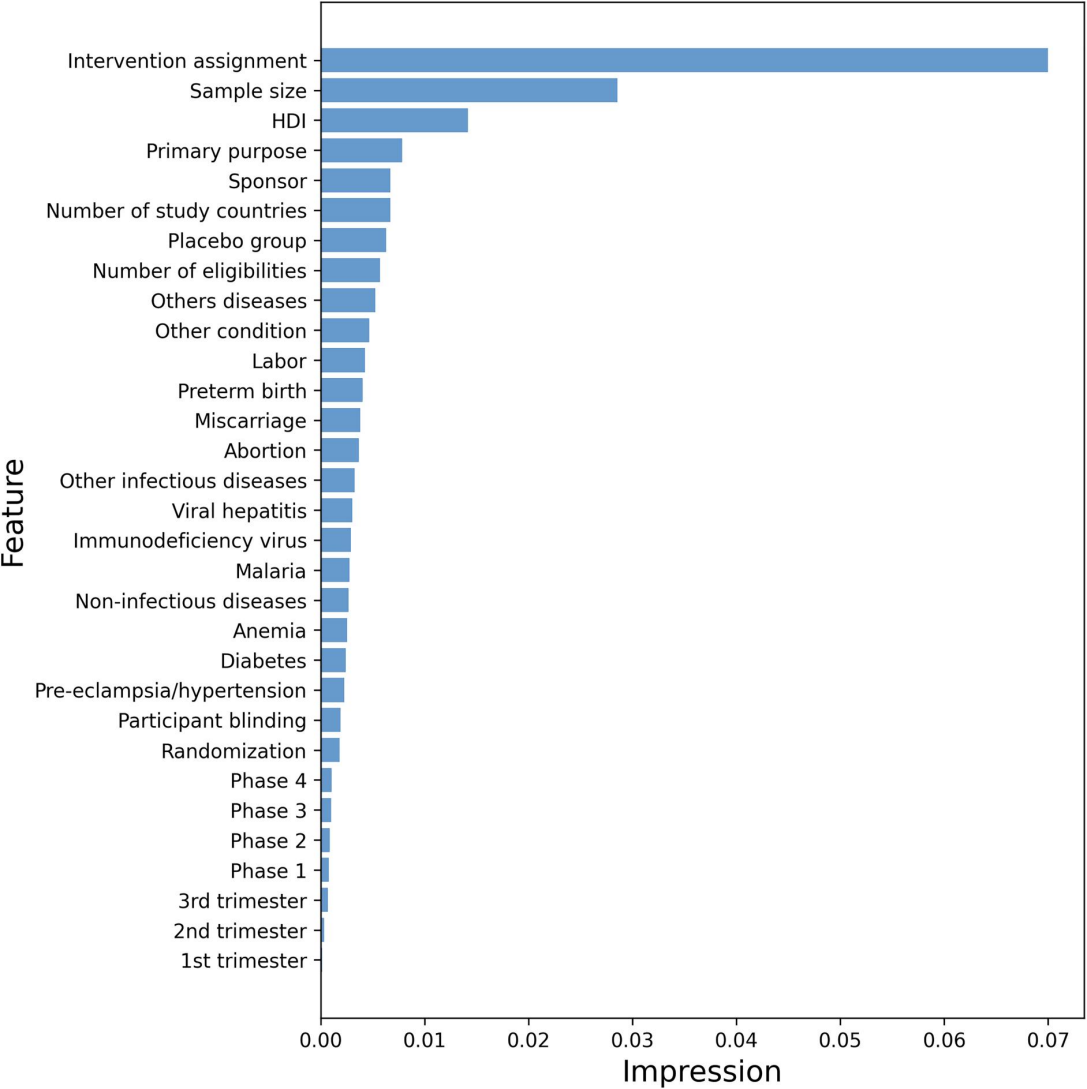
Effect of features on the concordance index (C‐index).

We compared the effect of all features on the concordance index (C‐index) (see Figure 2). A new C‐index was obtained by removing the specified features while keeping other values constant. The values in Figure 2 represent the difference between the initial test set C‐index and the new C‐index. As can be seen from the graphs, the intervention allocation, human development inde (HDI) and sample size had the greatest impact on the final results, and the model performance changed significantly after the removal of these features.

## 3 RELATED WORKS

### 3.1 Clinical trial termination prediction

Many researchers have studied clinical trial termination, identified characteristics associated with clinical trial termination and predicted clinical trial completion. Using a Random Forest algorithm, Follet et al. identified characteristics associated with clinical trial termination [4]. However, the predictive performance of the model was not excellent (sensitivity = 0.56; accuracy = 0.71 and F1 score = 0.12) [4]. Geletta et al. [3] also used the Random Forest algorithm. Elkin et al. [10] achieved a final area under the curve (AUC) prediction performance of 0.73 by identifying features associated with clinical trial termination and by training classifiers (neural network, Random Forest, XGBoost and logistic regression).

Although a number of researchers have studied clinical trial completion, none have achieved high accuracy and fewer have predicted clinical trial completion in pregnant women. The use of survival analysis models to study clinical trial completion continues to face many challenges.

### 3.2 Cox proportional hazards model

The traditional CPH model assumes that the ratio of an individual’s hazards function to the baseline risk function of the population is a constant that does not change over time, and assumes a linear relationship between the model parameters and the predictor variables, using linkage functions to predict individual hazards functions. Building on traditional survival analysis models, some newer survival analysis methods have made improvements mainly in model parameter optimization methods or model penalty terms. In 1995 Faraggi and Simon first proposed an extension of CPH with neural networks, where they used the output of a single hidden layer MLP to replace the linear combination of covariates in the CPH, providing a non‐linear extension of the CPH model with a non‐linear extension [14]. However, later studies found that this model usually failed to outperform the CPH model [15, 16]. This may be due to the limitations of the computing power and the training methods of neural networks at that time.

In 2018 Katzman et al. revisited these models within the framework of deep learning and proposed DeepSurv, a CPH model based on novel neural network extensions, but the model is still constrained by the proportional risk assumption [17]. Based on these studies, in 2020 Kvamme et al. proposed new event‐time forecasting methods, Cox‐CC, Cox‐Time, by extending the CPH with neural networks [18]. These two methods have the flexibility of neural networks while modeling event time continuously, and are highly competitive in survival analysis.

Although CPH models are currently making good progress in predicting survival analysis, for datasets with sparse feature spaces such as clinical trials they may lead to overfitting of the model, as well as to reduction in the actual prediction accuracy of the model and poor model robustness.

### 3.3 Denoising autoencoder (DAE)

In 2008, Bengio proposed that when an unsupervised approach is used to pre‐train the weights of a deep network in layers, random noise can be introduced into the visual layer of the network (i.e. the input layer of the data) to learn more robust features, a method called DAE [19]. DAE reconstructs clean (repaired) inputs from partially corrupted (damaged) inputs to force the hidden layer of the autoencoder to capture more robust features [13].

To prevent overfitting, Zhou et al. used DAE for online incremental feature learning, which adds new features to minimize the residuals of the objective function and merges similar features to obtain a compact feature representation [20]. To reduce the effect of noise, Chai et al. used features compressed by a DAE to estimate the risk of mortality of cancer patients [21]. To enable further accuracy in medical image analysis, Zhong et al. used the DAE based on convolutional neural networks to denoise and classify CT images of brain tumors [22]. Gu et al. generated composite features from multi‐omics data in cancer genome mapping by using denoising autoencoders to robustly identify ovarian cancer subtypes [16]. Xiao et al. used regions of interest in lung nodules as input data and extracted features using the DAE ResNet‐18 to predict the malignant phenotype of lung nodules [14]. To extract meaningful and more robust features from lung ADC data relevant to survival prediction, Wang et al. used a DAE to discover molecular features consisting of multiple genes of ADC [12].

To solve the problem of sparse dataset7 used for survival analysis, some researchers have combined the DAE with survival analysis models. Liu and Ping developed two unsupervised DAE to jointly extract deep features from breast cancer genomic data and combined them with Cox models to evaluate the association between each feature and overall patient survival [17]. Atlam et al. proposed the Deep_Cox_COVID_19 system based on Cox regression. Deep_Cox_COVID_19 first applied the autoencoder to the data to reconstruct features and then applied the Cox regression algorithm for survival analysis studies to improve prediction accuracy and precision [15].

Although DAE currently achieve good results in extracting features from medical disease datasets and using Cox models for survival analysis prediction, few have combined noise‐reducing autoencoders with CPH models for survival analysis prediction of clinical trial completion. There are still many challenges in solving the problem of sparse clinical trial dataset of pregnant women based on using DAE and predicting clinical trial completion of pregnant women with CPH models.

## 4 CONCLUSIONS

Predicting clinical trial completion in advance to avoid unnecessary waste of resources is very meaningful. In this paper, we propose a new survival analysis model by combining DAE with DeepSurv for prediction of the completion of clinical trials involving pregnant women. The model solves the problem of model overfitting due to the sparsity of features in the clinical trial dataset. Experimental results show that our model improves the accuracy and robustness of the model compared to DeepSurv. In addition, we point out the experimental parameters that affect the probability of clinical trial termination, so that subsequent researchers can easily adjust the clinical trial protocol in advance, and so more effectively avoid early clinical trial termination.

Although the model proposed in the paper showed good performance in predicting the completion of clinical trials involving pregnant women, there are still some issues that need further investigation. For example, the improved training accuracy of DAE‐DSR compared to DeepSurv is not very significant, which may be related to the size of the dataset itself and the level of confusion of the dataset, and thus further experiments on larger sample sizes and more complex databases may still be needed.

Several limitations exist in datasets due to the nature of registry‐based research. First, we did not analyze all clinical studies conducted on pregnant women but analyzed a subset registered at ClinicalTrials.gov. Second, because ClinicalTrials.gov is a US registry, it contains many studies conducted in North America. If another registry were to be used, there might be differences in study locations and study characteristics, such as target medical conditions and trimesters. Last, the quality of the data entered into the database depends on the study investigators or sponsors.

## 5 MATERIALS AND METHODS

**FIGURE 3:**
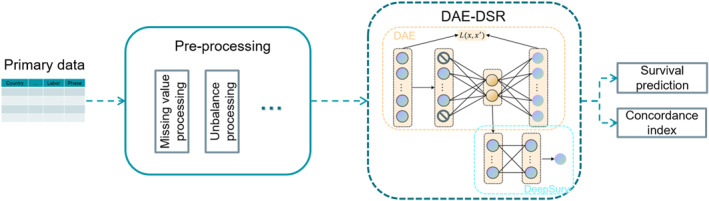
Denoising autoencoder and deep survival regression model workflow diagram.

The model proposed in the paper aims to address the problem of making DeepSurv maintain a reliable prediction level despite sparse data. This section describes the proposed model and the model structure is shown in Figure 3. The model can broadly be divided into two modules: preprocessing and DAE‐DSR. Before training, the data is preprocessed and the processed data features are input into DAE‐DSR for survival prediction. The details of the steps involved in the proposed framework are presented in the following subsections.

### 5.1 Datasets

The dataset used [9] was from clinical trials involving pregnant women, and collected from ClinicalTrials.gov, one of the largest publicly available databases of clinical trials. It allows researchers to search for information on registered clinical studies and to keep up to date with the conduct of clinical trials in relevant subject areas. The data is collected from clinical studies initiated on the website between 1 January 2009 and 31 December 2018. The 2002 Council for International Organizations of Medical Sciences guidelines state that pregnant women should be presumed to be eligible for participation in biomedical research [23], and in 2018 the US Food and Drug Administration announced draft guidelines for scientific and ethical considerations for including pregnant women in clinical trials [24]. Thus the use of data involving clinical trials on pregnant women are ethical for modeling. The relevant description of the dataset is shown in Table 2. By searching the website for the keywords “pregnancy”, “maternal”, “antenatal” and “gestation,” and requesting at least one drug to be used in the intervention studies, the data from 819 clinical trials were obtained. Of these, data with a recruitment status field of “completed” on the ClinicalTrials.gov website were considered to be normal study completion, that is, complete data, while other recruitment statuses were considered review data. Time was defined as the period from the study start date to the study completion date or the date of the last posted update, whichever came first. Characteristics of the dataset included preplanned sample size (categorized into quartiles: 0 ≤ *n* < 80, 80 ≤ *n* < 150, 150 ≤ *n* < 329 and *n* ≥ 329), stage of pregnancy, number of study countries, HDIxHDI of study countries, study stage, sponsors, randomization, intervention allocation, subject blinding, primary aim, placebo group, target medical status and number of eligibles. In addition, the dataset was randomly divided into a training set (80%) and a test set (20%). This paper uses the model created to make predictions about whether the clinical trial data will terminate normally and to give the features that will have the most impact on the results.

**TABLE 2:** Description of the datasets.

	Notes
Origin	ClinicalTrials.gov
Searching keywords	“Pregnancy”, “maternal”, “antenatal” and “gestation”
Size	819
Characteristics of the dataset	Preplanned sample size, stage of pregnancy, number of study countries, HDI, study stage, sponsors, randomization, intervention allocation, subject blinding, primary aim, placebo group, target medical status and number of eligibles
Training set: validation set	8:2
Date type	Float32

*Note*: Preplanned sample size, categorized into quartiles: 0 ≤ *n* < 80, 80 ≤ *n* < 150, 150 ≤ *n* < 329, and *n* ≥ 329.

Abbreviation: HDI, human development index.

After reading the dataset, one noted that it included no duplicate invalid data. The dataset had 33 covariates: we normalized the five numerical covariates and left the remaining binary covariates unchanged. In addition, all feature values were converted to a uniform data type, here we chose the float32 type. The dataset used to train the model was divided into a training set and a validation set in the ratio of 8:2, from which the time and event columns were extracted as the label values respectively. Finally, the separate training and validation sets were combined with their label values into a tuple of data for subsequent training of the model.

### 5.2 Experimental setup

We implemented DAE‐DSR using the Pytorch framework and the pycox package. Encoder E and decoder D each had two hidden layers, where the first hidden layer had 32 nodes and the second had 16. ReLu was used as a non‐linear activation function between each of the two layers. The survival analysis network layer consisted of a simple MLP with a single hidden layer containing a ReLu activation layer, a batch normalization layer, and a dropout (value 0.1) to address the overfitting problem. In addition, we used Gaussian noise to add noise to the input. The batch size used for network training was 256 and the Adam optimizer had a learning rate of 0.01. In the loss function, we set *α* to 0.6 and *β* to 0.4. We used the concordance index (C‐index) [25] to evaluate our model and compare it with some previous studies.

### 5.3 Data preprocessing

Clinical trial data are often not directly useable for prediction tasks due to noise, incompleteness, and inconsistency. As a result, data preprocessing is performed in order to extract valid features from the data before data analysis is undertaken. Data preprocessing includes missing value processing, data normalization processing, data imbalance processing, and so on. As a result, data pre‐processing is required before applying the prediction model. The steps involved in preprocessing are as follows:

Step 1: Reading of the Comma‐Separated Values file using Pandas [26] and removal of duplicate and invalid samples from the original data.
Step 2: Missing values processing. Feature columns with missing values greater than 80% were removed from the data processed in the previous step. The KNN algorithm was used to interpolate the missing values of the remaining data. The algorithm filled in the missing value samples by using the *k* most adjacent samples in the feature space.
Step 3: Data normalization. Some categorical fields were in text form and needed to be normalized and converted to integers. In survival analysis, censored data is a crucial aspect of the analytical model. Target events are classified as completed and censored. The binary classification fields are generally converted into binary numbers, that is, 0 or 1.
Step 4: Data imbalance processing. The SMOTE algorithm from the imblearn [27] package was used to deal with the data imbalance problem. This algorithm differed from the oversampling technique based on simple replication of minority class samples. Instead, it synthesized new minority class samples by linear interpolation of existing minority class samples, thus effectively solving the data imbalance problem.

### 5.4 DAE‐DSR model

**FIGURE 4:**
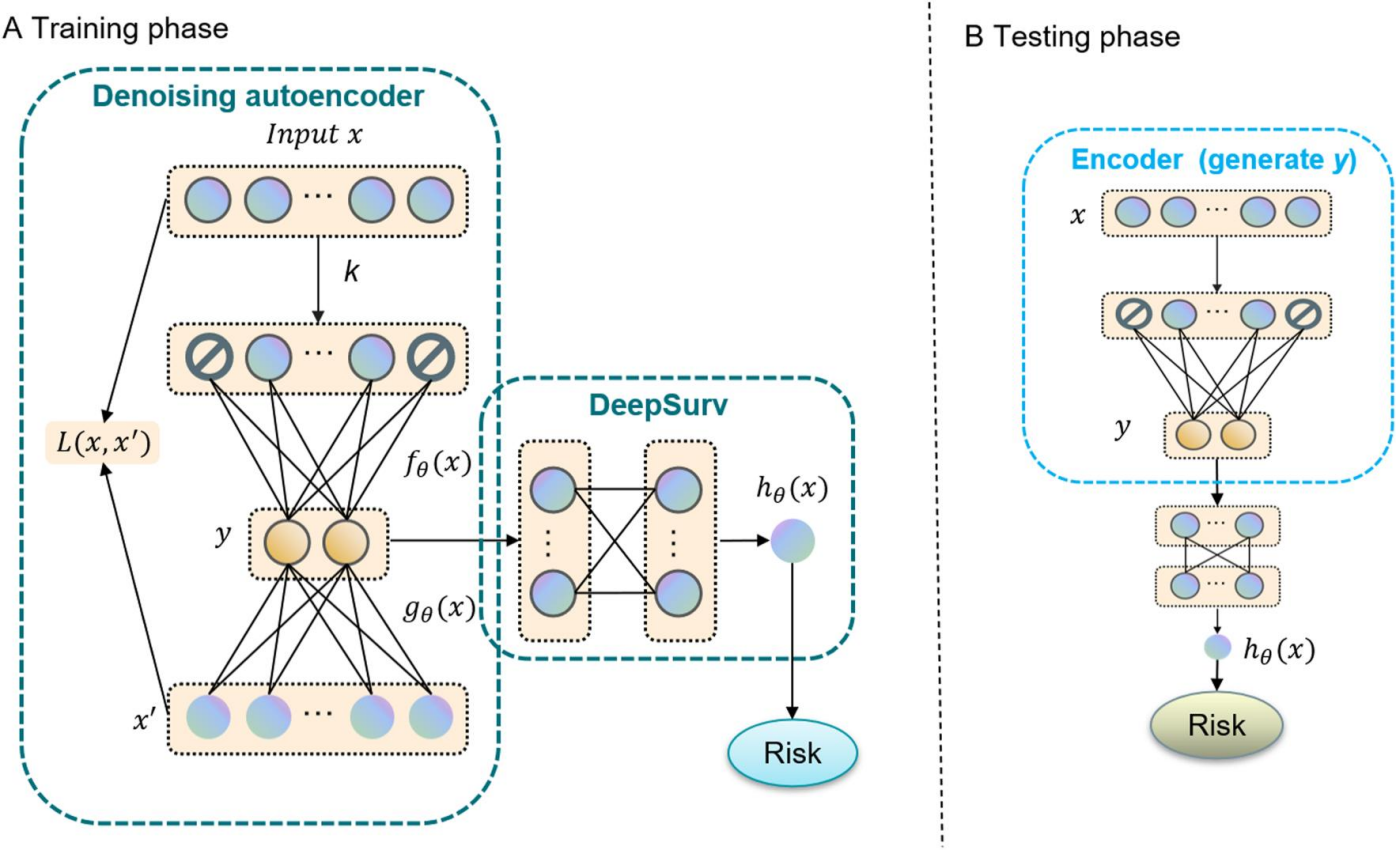
Schematic diagram of the denoising autoencoder and deep survival regression architecture. It consisted of a noise‐reducing autoencoder and DeepSurv. DAE was used to generate a robust representation of the data and DeepSurv predicted the likelihood of clinical trial completion.

DAE‐DSR consisted of a noise reduction autoencoder and DeepSurv, as shown in Figure 4. The data *x* obtained in the preprocessing phase was used as input to the DAE‐DSR model, and the data was passed through the DAE to generate robust features *y*, which were used as input features for training in DeepSurv. In the test phase, the encoder generated a compressed feature representation directly and passed it to the DeepSurv model. Its two main components are described below.

#### 5.4.1 Denoising module

The autoencoder uses unsupervised learning and does not require the labeling of the training samples. *x*=(*x*_1_, …,*x*_*n*_) is used as the input vector, which is mapped in the encoder as a potential feature vector *y* by *y* = *f*_*θ*_(*x*) and then decoded as *x* ^′^ by *y* = *g*_*θ*_(*x*), which is the same size as *x*. The purpose of AE is to adjust the network parameters so that the final output *x* ^′^ is as close as possible to the original input *x*. Depending on the assumed distribution of the input data, there are several error calculations, such as the mean square error:

(1) $$\begin{array}{c} L\left(x,x^{\prime }\right)={\left\|x-x^{\prime }\right\|}^{2} \end{array}$$

DAE is based on autoencoder, where noise is added to the input data (the input layer of the network) in order to prevent overfitting problems, which also makes the encoder more robust and thus enhances the generalization of the model. The feature after adding noise is $\tilde{x}=k\left(\tilde{x}\;|\;x\right)$.

In this paper, clinical trial features are added to the noise by $k\left(\tilde{x}\;|\;x\right)$, and $\tilde{x}$ is then used as input to the encoder for training. The decoder output *x* ^′^ is iterated with the original *x* for error, and the model is optimally updated through a process of minimizing the loss function.

#### 5.4.2 Survival analysis module

The main purpose of survival analysis is to investigate the relationship between the covariate (independent variable) *X* and the observed outcome, that is, the survival function *S(t, X)*, and a very important element of survival analysis is to explore the risk factors affecting survival time or survival rate [18], which is usually expressed as the product of the baseline risk rate function and the corresponding covariate function, as in Equation (2).

(2) $$\begin{array}{c} h\left(t,X\right)=h_{0}\left(t\right)\cdot f\left(X\right) \end{array}$$

where *h*(*t*,*X*) is the risk rate function at time *t*, also known as the instantaneous mortality rate. *h*_0_(*t*) is the baseline risk rate function at time *t*, that is, the risk rate function at time *t* when all covariates take on the value 0; *f*(*X*) is the covariate function.

DeepSurv [18] uses a MLP, similar to the Faraggi‐Simon network, that solves survival analysis tasks by training a neural network to approximate *h*(*x*), and that is able to model non‐linear relationships in the hazard function. It is based on the CPH model with the following refinements, as in Equation (3).

(3) $$\begin{array}{c} \lambda \left(t\;|\;x\right)=\lambda_{0}\left(t\right)\cdot \;e^{h_{\theta }\left(x\right)} \end{array}$$

where *θ* is the learning parameter of the neural network. DeepSurv uses a MLP to estimate *h*_*θ*_(*x*). The robust features obtained from the encoder are fed into DeepSurv along with the temporal event labels for survival prediction.

#### 5.4.3 Loss function

DAE‐DSR trains DAE and DeepSurv jointly, and we use the mean squared error as the reconstruction loss for the noise reduction autoencoder and we use the Breslow approximation with negative log‐likelihood as the loss for risk prediction. For the joint model DAE‐DSR, we combine the reconstruction loss *l*_DAE_ with the surviving loss *l*_DeepSurv_, using a weighted linear sum [28] to combine the two loss functions to obtain the loss of the model, as in Equation (4).

(4) $$\begin{array}{cc} l=\alpha l_{DeepSurv}+\gamma l_{DAE} & =\gamma \sum_{i=1}^{n}{\left(x_{i}-g_{\theta }\left(f_{\theta }\left(\tilde{x}\;\right)\right)\right)}^{2}-\alpha \sum_{i,E_{i}=1}\left({\hat{h}}_{\theta }\left(x\right)-\log\sum_{j\epsilon R\left(T_{i}\right)}e^{{\hat{h}}_{\theta }\left(x_{j}\right)}\right) \end{array}$$

*T*_*i*_, *E*_*i*_, and *x*_*i*_ denote the event time, event metric, and the *i*th baseline data respectively. *E*_*i*_ = 1 denotes the set of patients to which the event can be observed to occur, and the risk set *R*(*t*) = {*i*:*T*_*i*_ ≥ *t*} denotes the set of patients still at risk at moment *t*. *α* and *γ* are used to calibrate the contribution of each term to the total loss, that is, to control for the loss of survival prediction and input reconstruction balance. During training, *α* and *γ* are considered hyperparameters in the network, and as the parameter *α* decreases, the weight of the corresponding target loss *l*_CoxPH_ is reduced. For ease of use, *γ* is considered as 1−*α* during training.

### 5.5 Evaluation criteria

#### 5.5.1 Concordance index

The concordance index, also known as C‐index [29] is one of the most frequently used discriminative measurement tools in survival analysis. The C‐index is a broadening of the AUC, which is defined as the ratio of concordant pairings to all similar pairs, and can account for censored data. It can be expressed as follows:

(5) $$\begin{array}{c} C=\frac{\sum_{i,j}l\left[T_{i}<T_{j}\right]\cdot l\left[{\hat{T}}_{i}<{\hat{T}}_{j}\right]\cdot \delta_{i}}{\sum_{i,j}l\left[T_{i}<T_{j}\right]\cdot \delta_{i}} \end{array}$$

*δ*_*i*_ = 1 if *T*_*i*_ can be observed, 0 otherwise. *T*_*i*_ is the risk score, and C‐index = 1 shows optimal model results in the same way that AUC does.

#### 5.5.2 Brier score

The BS is a discriminant and calibrated measure of model estimation used to evaluate the precision of the predicted survival function at a certain time *t*. BS shows the average squared difference between the predicted survival probability and the observed survival state, that is, $\text{BS}=1/N\sum_{i}{\left(y_{i}-\hat{p_{i}}\right)}^{2}$, with values ranging from is 0 to 1, where 0 is optimal. By weighting the scores according to the inverse censoring distribution, Graf et al. [30] expanded the BS to take right censoring into account:

(6) $$\begin{array}{c} \text{BS}\left(t\right)=\frac{1}{N}\sum_{i=1}^{N}[\frac{\hat{S}{\left(t\;|\;x_{i}\right)}^{2}l\left\{T_{i}\leq t,D_{i}=1\right\}}{\hat{G}\left(T_{i}\right)}+\frac{{\left(1-\hat{S}\left(t\;|\;x_{i}\right)\right)}^{2}l\left\{T_{i}>t\right\}}{\hat{G}\left(t\right)}] \end{array}$$

*N* is the quantity of observations. Using Kaplan‐Meier calculations, $\hat{G}\left(t\right)$ is an estimate of the censoring survival function under the assumption that censoring and survival times are independent of each other.

## AUTHOR CONTRIBUTIONS

**Huamei Qi**: Formal analysis; funding acquisition; supervision; writing – review and editing. **Wenhui Yang**: Data curation; formal analysis; methodology; software; visualization; writing – original draft preparation. **Wenqin Zou**: Data curation; formal analysis; software; writing – original draft preparation. **Yuxuan Hu**: Conceptualization; funding acquisition; resources.

## CONFLICT OF INTEREST STATEMENT

The authors Huamei Qi, Wenhui Yang, Wenqin Zou, and Yuxuan Hu declare that they have no conflict of interest or financial conflicts to disclose.

## ETHICS STATEMENT

This article does not contain any studies with human or animal subjects performed by any of the authors.
